# Polish physicians’ cooperation with the pharmaceutical industry and its potential impact on public health

**DOI:** 10.1371/journal.pone.0184862

**Published:** 2017-09-19

**Authors:** Marta Makowska

**Affiliations:** Faculty of Social Sciences, Warsaw University of Life Sciences, Warsaw, Poland; National Academy of Medical Sciences, NEPAL

## Abstract

**Objective:**

This article aims to describe how Polish physicians cooperate with the pharmaceutical industry and show how this relationship may pose a threat to public health.

**Methods:**

It considers the results of an online survey of 379 physicians. The survey was hosted by surveymonkey.com with links from a Polish physicians’ website (Medycyna Praktyczna) between 29 October 2013 and 31 December 2013. The sample was purposive, respondents having to be physicians working in Poland.

**Results:**

The majority of respondents (96.8%) said that they had talked with pharmaceutical sales representatives (PSRs) in their practice, with 85% saying that they had had regular contact with them. Despite the existing legal ban in Poland, 35% of respondents admitted that they had usually met with PSRs in their office during working hours. As many as 81.8% of surveyed doctors said that they had taken part in an educational meeting organized by the pharmaceutical industry at least once during the 12 months preceding the study. A majority of the respondents (72.3%) said they trusted the information provided by PSRs. Over one third of respondents (36.4%) claimed that Polish doctors accepted gifts of a type that they should not accept according to Polish law.

**Conclusions:**

The study showed that Polish physicians cooperate in different ways with pharmaceutical companies and have frequent contact with them. This can influence their knowledge and doctors whose knowledge of drugs is based mainly on information from pharmaceutical industry materials may prescribe medicines in a biased way, possibly exposing their patients to sub-optimal treatments and burdening both their patients and the state budget with unnecessary costs. Lack of trust in doctors and pharmaceutical companies have other implications too: there may be a decline of faith in the efficacy of therapy and patients may be encouraged to engage in self-diagnosis and self-treatment. For these reasons it is necessary to increase transparency and strengthen the ethical guidelines surrounding the physician–pharmaceutical industry relationship in Poland. The present findings also have implications for public health policy.

## Introduction

A physician’s social role requires that people have confidence in them. For many years, doctors’ relationships with the pharmaceutical industry, which sponsors the education of doctors, provides meals and gives them gifts, grants and other benefits, has made patients doubt whether doctors always put their best interests first [1]. Various studies have confirmed these fears, the acceptance of benefits in various forms from pharmaceutical companies influencing the medicines physicians prescribe [2–6], and doctors obtaining the greatest benefits from corporations tending to prescribe the largest number of innovative medicines [6]. Also, physicians have been shown to trust information from the pharmaceutical industry [7,8], although it has been proven that the industry often communicates inaccurate data [7,9]. As a result, pharmaceutical marketing can lead to sub-optimal prescribing of medicines (excessive prescribing, and prescribing of medicines which are sub-optimal in terms of their effects on patient health or financial costs to the patient or state), self-diagnosis and self-treatment, pharmaceuticalization, and the medicalization of society [1,10].

Appropriate transparent relationships between pharmaceutical companies and doctors are crucial for public health, and cooperation between the two parties is necessary because the industry produces new drugs. Therefore the industry has an obligation to inform the medical world about the creation and effects of their products. However, doctors’ relationships with the pharmaceutical industry often lead to a conflict of interest: a physician who should act in the best interest of their patient is tempted by gifts, conferences in attractive places and meals from pharmaceutical companies to put business interests or their own interests over the interests of the patient. Doctors who are faced with a conflict of interest may prescribe drugs sub-optimally for their patients (drugs of a higher price, weaker effectiveness or efficiency). Often such biases do not arise from conscious processes, physicians denying that there is any relationship between industry gift-giving and their professional objectivity [2,7,11,12]. However, even the acceptance of small gifts such as a pen can lead to reciprocity [13,14]. The more valuable the gift, the more physicians are conscious of reciprocity, larger gifts being more likely to cause ethical dilemmas and bring into question the issue of whether or not a gift may influence a physician [13]. Research has indicated that almost all doctors accept small gifts such as pens, mugs, medication samples and meals from pharmaceutical companies [6,11,12]. Far fewer doctors receive more valuable gifts such as royalties for oral presentations at conferences and fully sponsored educational meetings [7,11,12,15].

The Polish pharmaceutical market is the largest in Central and Eastern Europe and the sixth largest in Europe [16]. The value of the Polish market has been growing for many years, its value in 2015 reaching PLN 29.9 billion, an increase of 4.8% compared to the preceding year [17]. This increase in size is attributable to the increasing affluence of Polish society, aging of the population, the development of new medicines, and a growing incidence of civilization diseases. The increasing value of the market has also contributed to an increase in pharmaceutical marketing directed at both patients and healthcare professionals, prescribing physicians constituting the most important target with respect to industry profits. Pharmaceutical companies’ methods of influencing Polish doctors are the same as those used in other countries. In accordance with relationship marketing theory, first contacts with physicians are established while they are still studying [18]. The industry offers gifts, meals, invitations to educational meetings, sponsors conferences and travel, and pays for consultations and speeches. To date, though pharmaceutical marketing directed at physicians is conducted on a large scale, little research on the cooperation of doctors or healthcare institutions with pharmaceutical companies has been done in Poland [8,19,20]. In 2011, 6429 PSRs worked in Poland, with one representative for every 25 physicians [18].

Ethical questions about cooperation between physicians and the pharmaceutical industry were raised in the Western world during the 1990s. In Poland this was a time of socio-economic transformation and the first multinational pharmaceutical companies entered the market using well developed marketing strategies. When PSRs first started to visit physicians they were well treated by doctors, and meetings were perceived as a privilege by physicians. Over time, however, these visits began to lose their importance, their frequency coming to be perceived as bothersome by both the medical fraternity and patients. Polish pharmaceutical law started to be more and more restrictive with respect to the nature of cooperation, due both to Polish accession to the European Union in 2004 and to changes in perceptions of these relationships. Currently, Polish law is perceived as strict in the area of physician–pharmaceutical industry cooperation [18]. For example, regulations state that a physician cannot receive gifts which are worth more than 100 PLN/24 Euros, or gifts which are unconnected with a physician’s practice [21], and doctors cannot meet with pharmaceutical sales representatives during office hours [22].

The Polish health care system is centralized and based on mandatory health insurance subsidized from the state budget [23]. This causes the system to be underfinanced. Low wages (66,500 PLN/ 15,830 Euros per year [24]) are driving physicians not only to look for better jobs abroad [25], but may also be leading to greater willingness to take gifts from pharmaceutical companies, and to attend conferences, presentations and educational meetings organized by the industry as many doctors cannot pay for themselves [8,26].

Patients’ awareness of physician–pharmaceutical industry cooperation can result in a loss of confidence in doctors [1,27,28]. Research has shown that only 36% of Polish people consider doctors to be reliable and honest [29], and the prestige of the medical profession is decreasing [30]. This is important because a lack of trust in doctors can affect patients’ health in many ways: if they are unsure whether a prescribed drug is actually the best one for them, they may replace it under the guidance of a pharmacist who is unaware of the full history of their ailment; they may not buy the prescribed medicaments; they may not use medicaments as directed, or take them without believing that they will actually help; they may self-diagnose and self-medicate, which can have undesirable effects.

Doubts about the ethics of physicians’ cooperation with the pharmaceutical industry and cases such as the withdrawal of Vioxx [31] have also undermined confidence in drug producers. People have started to lose the belief that medications are safe, efficient, and constitute good value. This has led to an increase in the popularity of anti-vaccine and anti-psychiatry movements [18].

Some countries have introduced strict restrictions on cooperation between pharmaceutical companies and healthcare practitioners. In recent years, great emphasis has been placed on greater transparency in such relationships. For example, the United States’ introduction of the Physician Payment Sunshine Act has forced pharmaceutical companies to publicize information about payments or other gifts to physicians if their value exceeds $10 or if they total $100 or more in a calendar year. Also, information concerning the provision of any medical instruments exceeding a total value of $100 has to be publicized [15]. Similar legal regulations requiring companies to disclose such data have also been introduced in France and Slovakia [32]. However, although there are strict pharmaceutical marketing laws, in Poland there are no such legal obligations. Rather, the industry is self-regulating, and INFARMA (The Employers' Association of Innovative Pharmaceutical Companies) produced a report in June 2016 using data from members who signed the ‘Transparency Code’ revealing how much money they spent on working with health organizations and representatives of the medical profession in 2015. This report showed that 107.6 million PLN/ 25.5 million Euros was allocated to various benefits for medical professionals. On average, any particular doctor obtained benefits worth 2772 PLN/656 Euros [33]. It is worthy of note that, because it is not a legal requirement, only 22% of doctors agreed to disclose their connections with the industry.

The main aim of study presented below is to describe how Polish physicians cooperate with the pharmaceutical industry. The purpose of research was to shed light on the research question: in what ways do Polish physicians cooperate with the pharmaceutical industry and what are the reasons for this? The implications of the answer to this question for public health is discussed subsequently.

## Materials and methods

The study was conducted using an online survey placed on the surveymonkey.com portal. The survey was conducted from 29 October 2013 to 31 December 2013. The sample was purposive, respondents having to be physicians working in Poland. Information about the survey and a link to it was placed on the Kurier Medycyny Praktycznej portal (one of the most popular portals aimed at medical professionals), on the Facebook boards of Young Doctors and Young Doctors Wielkopolska, and posted on goldenline.pl (the Polish version of Linkedin), which is aimed at young doctors. The main disadvantage of this type of research is that participation was voluntary and likely to have included a disproportionate number of doctors who were keen on using the Internet in general and social media in particular. Thus, the results of the study may not be generalizable to the entire population of doctors in Poland. The project aimed to obtain data from at least 350 physicians, based on a past research from 2008, but in the event 379 correctly completed questionnaires were obtained.

Questionnaire completion took around 10 to 15 minutes. The questionnaire contained 25 questions about interactions between the pharmaceutical industry and physicians, and seven demographic questions. Data for 112 independent variables was obtained, and analyzed using PS Imago 4. The questionnaire (S1 File) and dataset are freely available at Figshare (DOI: https://doi.org/10.6084/m9.figshare.4924226.v1). Frequencies were calculated for each variable, and descriptive statistics were derived for numeric variables. Inferential analyses took the form of (non-parametric) tests such as Pearson's chi-square tests of association, Mann–Whitney U tests and Kruskal-Wallis tests. In this article only the most interesting and important results of the study are presented.

The questionnaire’s content validity was examined to ensure that responses would provide data to allow all the project’s research questions to be answered. A pilot study was conducted with ten doctors to iron out any problems with the questionnaire and determine whether the survey include all the questions needed to measure all concepts. The reliability of participants’ responses was examined by ensuring the logical coherence of responses on each questionnaire (e.g., by checking that age and seniority data were logically consistent, and that people had not said that they started a job in the medical profession before the age of 23: it is unlikely that such people would have finished medical school at such a young age). All data from unfinished or logically dubious questionnaires were excluded from analysis, thus screening out non-physicians, it being reasonable to assume that such people would not have been able to successfully complete the survey in a coherent manner. Additionally, questionnaires completed by doctors currently working abroad, long since retired or unemployed were excluded since the study aimed to investigate only the current situation in Poland.

No Institutional Review Board consent was obtained since Warsaw University of Life Sciences lacks a social science research board. However, care was taken to minimize ethical problems, participants submitting their questionnaires anonymously with no identifying information being collected, the survey being online, and being completed voluntarily by physicians.

## Results

### Contacts with pharmaceutical sales representatives

The study showed that 96.8% of doctors had at least one contact with a PSR in the course of their practice, with 85.0% of physicians meeting them regularly. Statistics relating to various socio-demographic characteristics of the sample and whether or not physicians usually met with PSRs are presented in Table 1.

**Table 1:** Socio-demographic characteristics and different aspects of cooperation with PSRs.

Sample characteristic	Usually met with PSR*	n	Total	n
SEX				
Female	88.3%	196	60.4%	229
Male	80.0%	116	39.6%.	150
SENIORITY				
Less than 5 years	82.0%	41	15.6%	59
From 6 to 15 years	83.1%	74	24.1%	90
From 16 to 25 years	84.7%	84	26.5%	99
Over 26 years of seniority	87.2%	109	33.7%	126
PROFESSIONAL STATUS				
Intern and Resident	80.0%	32	12.4%	47
Non-specialist doctor and specialist doctor under the training	94.1%	32	10.0%	38
Specialist doctor	85.7%	239	73.9%	280
Other	64.3%	9	3.7%	14
WORKING PLACE (respondents could choose more than one answer)				
Public hospital	82.3%	153	51.2%	194
Non-public hospital	87.5%	21	6.6%	25
Public clinic	90.7%	98	28.8%	109
Non-public clinic	88.0%	139	42.5%	161
Office, clinic or hospital without a contract with the National Health Fund	83.2%	89	22.3%	109
Emergency	75.0%	9	3.4%	13
Other	66.7%	14	5.5%	21
NUMBER OF WORKING PLACES				
One	86.2%	175	55.6%	212
More than one	83.5%	137	44.4%	167
REPRESENTATIVE EXPERIENCE				
Worked as PSR	100%	15	4.5%	17
Not worked as PSR	84.4%	297	96.5%	362
PLACE OF DOMICILE				
Village and cities up to 200,000 residents	89.6%	173	52.2%	198
City with over 201,000 residents	79.9%	139	47.8%	181

Five chi-square tests and one Fisher’s exact test were used to test associations between whether or not people usually met with PSRs and various socio-demographic characteristics. The first test showed that there was a significant association between gender and meeting PSRs, χ^2^(1) = 4.7, p < .05 (see Table 2). Tests of significant differences (p < .05) between column proportions showed that females usually met PSRs more frequently than males (and conversely that males usually did not meet PSRs more than females).

**Table 2:** Observed counts (expected counts) and % within gender for the chi-square test of association between gender and meeting PSRs.

Meeting of PSR	Gender		Observed Total
	Female	Male	
**Usually meet**	196 (188.7) 88.3%	116 (123.3) 80.0%	312
**Usually do not meet**	26 (33.3) 11.7%	29 (21.7) 20.0%	55
**Observed Total**	222	145	367

Table 3 shows the association between professional status and meeting PSRs. A chi-square test showed a marginally significant association, χ^2^(3) = 7.8, p = .05. Tests between column proportions showed that doctors of ‘other’ professional status were significantly less likely to say that they usually met PSRs than both non-specialist doctors etc., and specialist doctors, and, as would be expected given this, that doctors of ‘other’ professional status were significantly more likely than non-specialist doctors etc. to say that they did not meet with PSRs, although here there was no significant difference between doctors in the ‘other’ group and specialist doctors. Physicians in the ‘other’ status category usually held managing positions or university positions with a higher degree.

**Table 3:** Observed counts (expected counts) and % within professional status for the chi-square test of association between professional status and meeting PSRs.

Professional status	Meeting of PSR				Observed Total
	Intern and Resident	Non-specialist doctor and specialist doctor in training	Specialist doctor	Other	
**Usually meet**	32 (34.0) 80%	32 (34.0) 94.1%	239 (237.2) 85.7%	9 (11.9) 64.3%	312
**Usually do not meet**	8 (6) 20%	2 (5.1) 5.9%	40 (41.8) 14.3%	5 (2.1) 35.7%	55
**Observed Total**	40	34	279	14	367

There was also a significant association between meeting PSRs and place of domicile, χ^2^(1) 6.8, p < .01. More physicians from villages, towns and small cities met regularly with PSRs than doctors from larger cities. Column proportion tests showed that doctors from villages and cities of up to 200,000 residents usually met PSRs significantly more than physicians from cities of over 201,000 residents, and physicians from the larger cities were significantly more likely to indicate that they usually did not meet with PSRs than those from villages and smaller cities (see Table 4).

**Table 4:** Observed counts (expected counts) and % within place of domicile for the chi-square test of association between place of domicile and meeting PSRs.

Place of domicile	Meeting of PSR		Observed Total
	Village and cities up to 200,000 residents	City with over 201,000 residents	
**Usually meet**	173 (164.1) 89.6%	139 (147.9) 79.9*%*	312
**Usually do not meet**	20 (28.9) 10.4*%*	35 (26.1) 20.1*%*	55
**Observed Total**	193	174	367

Note that other chi-square tests showed that there were no significant associations between meeting with PSRs and both seniority and number of working places, and also that a Fisher’s exact test (conducted because of low expected frequencies) showed no significant association between meeting with PSRs and having worked as a PSR.

### Reasons for meeting with PSRs

**Fig 1:**
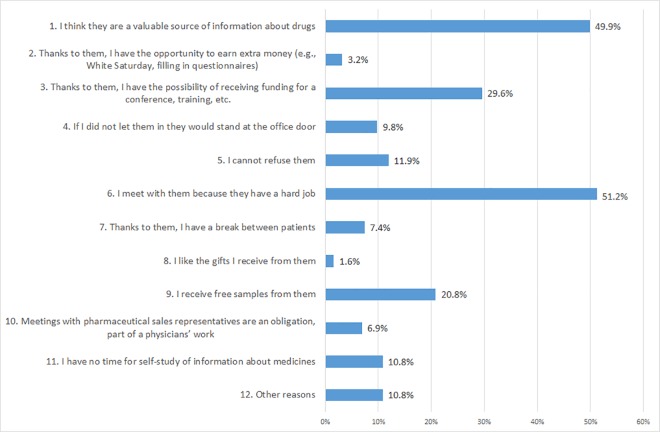
Reasons why physicians met with pharmaceutical sales representatives. Note. Percentages do not add up to 100% because physicians chose the three most important reasons.

The most common reason that doctors who met PSR’s gave for doing so was that they understood the difficulty of a PSR’s job (51.2%), indicating that most physicians met with PSRs because they felt pity for them. More alarmingly, 49.9% of respondents said that they met with representatives because they believed that the information provided by PSRs is valuable. The third most popular reason was the fact that PSRs might sponsor a conference or training place (29.6%). Only 1.6% of the surveyed physicians agreed that they liked the gifts offered by PSRs. More details of these data are presented in Fig 1.

On 1 December 2008 the Polish Minister of Health implemented a ban on PSRs visiting physicians during their working hours [22]. Despite this ban, 35% of doctors said they usually met with representatives in their offices during working hours, and an additional 31.8% said they met with PSRs in their offices, but only during breaks. On average, the physicians surveyed met with four PSRs per week. The length of the last meeting they had with a PSR was 9.5 minutes on average, although some doctors indicated their meeting lasted only one minute while one said it had lasted 45 minutes.

### Methods of cooperation

Besides meetings with PSRs, it was evident that the pharmaceutical industry tried to establish contacts with Polish doctors in other ways. A large number (86.8%) of physicians chose at least one form of cooperation, and for most of them (52.7%) it was restricted to one form. In the 12 months preceding the survey, the most common form (81.8%) was participating in conferences, presentations, or educational meetings organized by the industry.

Thirty seven percent of physicians chose at least one form of cooperation that was connected with the possibility of earning additional money. Of the surveyed physicians, 15.3% of respondents had been employed as speakers at a conference, presentation, or meeting and 15.6% had participated in studies concerned with the effectiveness of a drug (Phase IV trials—physicians received compensation from drug companies for completing questionnaires about patients’ reactions to medicines, such situations are often referred to as post-marketing surveillance trials). Also, 15.3% had participated in studies assessing the work of PSRs. Few doctors had taken part in examinations that were free for patients because they were sponsored by companies (known as White Sundays in Poland), and few (3.2%) had written or endorsed articles about a specific active substance or company’s drugs (see Table 5).

**Table 5:** Associations between doctors’ seniority and different methods of cooperation between Polish doctors and the pharmaceutical industry in the 12 months preceding the survey.

Means of cooperation	Less than 5 years	6–15 years	16–25 years	Over 26 years	Statistic parameters	Total
1. Participation as an audience member in a conference, presentation, meeting, or educational training organized by a pharmaceutical company.	80.5%	78.7%	84.5%	82.7%	χ^2^(3) 1.0, p = .79	**81.9%**
2. Participation as a presenter in a conference, presentation, meeting, or educational training organized by a pharmaceutical company.	7.3%	12.0%	17.9%	19.1%	χ^2^(3) 4.3, p = .23	**15.5%**
3. Participation in post-marketing examinations (Phase IV trials): completing a questionnaire about the reaction of patients to a medicine produced by a company.	7.3%	21.3%	21.4%	10.9%	χ^2^(3) 7.9, p < .05	**15.9%**
4. Participation in a marketing survey sponsored by a pharmaceutical company about the work of pharmaceutical sales representatives.	19.5%	14.7%	17.9%	12.7%	χ^2^(3) 1.5, p = .67	**15.5%**
5. Participation in free-for-patient examinations sponsored by a pharmaceutical company.	0.0%	5.3%	7.1%	4.5%	χ^2^(3) 5.0, p = .17 likelihood ratio	**4.9%**
6. Writing or endorsement of articles about a drug or active substance at the request of a pharmaceutical company.	2.4%	4.0%	7.1%	0.0%	χ^2^(3) = 10.5, p < .05 likelihood ratio	**3.2%**

Note. Total percentages do not add up to 100 because respondents could select all forms of cooperation engaged in during the previous 12 months.

With respect to forms of cooperation, four ordinary (Pearson’s) chi-square tests of association and two likelihood ratio chi-square tests were performed. There was a significant association between seniority and participation in post-marketing examinations, χ^2^(3) = 7.9, p < .05 (see Table 6). Here, while neither column proportion tests nor standardized residuals yielded significant results, the pattern for the largest standardized residuals was one whereby the two middle seniority groups had positive residuals for doctors saying that they participated in post-marketing examinations (z_resid_ = 1.2 for 6 to 15 years seniority, and z_resid_ = 1.3 for 16 to 25 years seniority), and the lowest and highest seniority groups had negative residuals for doctors saying that they participated in post-marketing examinations (z_resid_ = -1.4 for less than 5 years seniority, and z_resid_ = -1.3 for over 26 years seniority). Thus, members of the former two groups were slightly more likely to say that they participated in post-marketing examinations than would be expected if there were no association between seniority and participation, and the members of the latter two groups were slightly less likely to say that they participated in post-marketing examinations than would be expected if there were no association between seniority and participation.

**Table 6:** Observed counts (expected counts) and % within seniority for the chi-square test of association between seniority and participation in post-marketing examinations.

Participation in post-marketing examinations	Seniority				Observed Total
	Less than 5 years	From 6 to 15 years	From 16 to 25 years	Over 26 years	
**Yes**	3 (6.5) 7.3%	16 (11.9) 21.3%	18 (13.3) 21.4%	12 (17.4) 10.9%	49
**No**	38 (34.5) 92.7%	59 (63.1) 78.7%	66 (70.7) 76.6%	98 (92.6) 89.1%	261
**Observed Total**	41	75	84	110	310

A likelihood ratio chi-square test showed that there was a significant association between seniority and writing or endorsing articles about a drug or active substance at the request of a pharmaceutical company, χ^2^(3) = 10.5, p < .05 (see Table 7). Tests of differences in column proportions showed that doctors with 16 to 25 years’ experience and with 6 to 15 years’ experience had written or endorsed articles for pharmaceutical companies during the last 12 months significantly more often than those with more than 26 years seniority (and conversely those with seniority of over 26 years were significantly more likely to say that they had not written or endorsed articles than those with seniority from 16 to 25 years and 6 to 15 years). It is also worthy of note that all physicians reporting such activity were from cities of above 201,000 residents and that most of them (80%) were specialists.

**Table 7:** Observed counts (expected counts) and % within seniority for the likelihood ratio chi-square test of association between seniority and participation in writing or endorsing articles.

Writing or endorsement articles	Seniority				Observed Total
	Less than 5 years	From 6 to 15 years	From 16 to 25 years	Over 26 years	
**Yes**	1 (1.3) 2.4%	3 (2.4) 4.0%	6 (2.7) 7.1%	0 (3.5) 0%	10
**No**	40 (39.7) 97.6%	72 (72.6) 96.0%	78 (81.3) 92.9%	110 (106.5) 100%	300
**Observed Total**	41	75	84	110	310

Other tests showed that there were no significant associations between seniority and: participation as an audience member at a conference etc.; participation as a presenter at a conference etc.; participation in marketing surveys sponsored by the industry about the work of PSRs, and; participation in free patient examinations sponsored by a pharmaceutical company.

### Physicians’ trust in the information provided by pharmaceutical sales representatives

Overall 72.3% of doctors said that they had some trust in the information provided by PSRs, although only a small number (2.5%) said that they definitely trusted such information, with most physicians (69.8%) choosing the answer ‘somewhat trust’. Among physicians who did not trust PSRs as a source of information, the most popular reason for skepticism (52.3%) was that PSRs were considered to be salespersons with the main aim of ‘selling’ a drug. The second most common reason was the belief that pharmaceutical companies manipulated information about their medicines (38.4%).

### Physicians’ sources of knowledge

The idea of doctors obtaining knowledge about drugs from employees of pharmaceutical companies seems natural: who has greater knowledge of the advantages and disadvantages of a product than its producer? However, most companies prefer to share marketing materials with physicians rather than reliable information. Among the surveyed doctors, 47.2% declared that pharmaceutical companies were among their three most frequently used sources of information about medicines, such information being provided via leaflets, meetings organized by companies, or medical representatives. The most common ways of acquiring knowledge about drugs were medical journals (51.7%), conferences (44.6%) and the Internet (37.7%). In a 2008 study which I conducted, 67% of physicians indicated that one of their three most commonly used sources of information about drugs came from pharmaceutical companies. Since neither the present nor the previous study used completely representative samples, a direct comparison between them is not possible, however the difference is large enough to warrant emphasis.

### Drug samples

The majority of doctors (86.2%) said that they had received drug samples from PSRs, most of these (41.3% of doctors overall) declaring that they usually gave the samples to the poorest patients who could not afford medication, and 28.4% usually started therapy with the samples provided. As many as 22.1% of doctors said they took the samples for themselves or their family. Few (4.4%) said they did not do anything with them, simply having left the samples lying in their office, and 2.2% said that they gave them to other medical staff.

Almost all the surveyed doctors (99%) were unware that according to Polish law physicians may only accept a maximum of five samples of one drug per year from PSRs [21], with 57% thinking that they could accept fewer. Also, 61.1% of doctors disagreed with the statement: ‘A sample is a gift from a pharmaceutical company’.

### Doctors’ knowledge of various aspects of legal cooperation with pharmaceutical companies

Physicians were asked four questions about various aspects of legal cooperation with companies. The first was: ‘Does Polish law allow physicians to accept small gifts (e.g., pens, notepads) from PSRs?’ The second was: ‘Does Polish law allow physicians to accept expensive gifts related to their medical practice (e.g., a branded stethoscope) from PSRs?’ According to Polish pharmaceutical laws, doctors can accept a gift if its value does not exceed 100 PLN/24 Euros [21]. Therefore, doctors should have answered the first question positively and the second question negatively. The third question was: ‘Does Polish law allow physicians to meet with PSRs during working hours?’ (as previously mentioned, this is illegal.) The last question was: ‘Does Polish law allow PSRs to leave leaflets for prescription drugs for patients in a waiting room?’ In Poland, direct advertising of prescription drugs to customers is prohibited (therefore this question should have been answered negatively).

An index of doctors’ knowledge was created from the above four questions, one point being obtained for each correct answer. Results showed that 21.6% of doctors had ‘perfect knowledge’ (4 points), 35.9% had ‘good knowledge’ (3 points), 19.8% had ‘reasonable knowledge’ (2 points), 5.5% had ‘little knowledge’ (1 point), and as much as 17.2% of respondents answered all the questions incorrectly. This index was the dependent variable in the analyses reported in Table 8, where Mann-Whitney and Kruskal-Wallis tests were used to determine whether differences on the index existed for various socio-demographic independent variables.

**Table 8:** Differences in doctors’ knowledge indices across selected socio-demographic characteristics.

Sample characteristic	M	SD	Mean rank	Rank sum	Statistic parameters
SEX					
Female	2.5	1.3	199.1	45601.5	**Mann-Whitney,** U = 15083.5, p < .05
Male	2.2	1.4	176.0	26408.5	
SENIORITY					
Less than 5 years	1.8	1.5	152.1		**Kruskal–Wallis,** χ^2^(3) = 8.0, p < .05
From 6 to 15 years	2.5	1.4	193.3		
From 16 to 25 years	2.5	1.3	191.2		
Over 26 years of seniority	2.5	1.2	195.6		
PROFESIONAL STATUS					
Intern and Resident	2.0	1.4	149.5		**Kruskal–Wallis,** χ^2^(3) = 10.0,p < .05
Non-specialist doctor and specialist doctor under the training	2.3	1.3	180.9		
Specialist doctor	2.5	1.3	199.3		
Other	2.0	1.6	167.2		
NUMBER OF WORKING PLACES					
One	2.4	1.4	185.7	38448.5	**Mann-Whitney**, U = 16920.5,p = .875
More than one	2.4	1.3	187.4	30929.5	
REPRESENTATIVE EXPERIENCE					
Worked as PSR	2.7	1.2	213.4	3628	**Mann-Whitney**, U = 2679,p = .35
Not worked as PSR	2.4	1.3	188.9	68382	
PLACE OF DOMICILE					
Village and cities up to 200,000 residents	2.6	1.2	203.2	40239	**Mann-Whitney**, U = 153000, p < .05
City with over 201,000 residents	2.1	1.4	175.5	31771	

Table 8 shows that there were some significant differences between doctors’ knowledge indices across socio-demographic characteristics of respondents. There was a significant sex difference, females having better knowledge than males. Also doctors from villages and smaller cities had better knowledge than those from larger cities.

As Kruskal-Wallis tests indicated differences in knowledge for the seniority and professional status dependent variables, Mann-Whitney tests (see Table 9 and Table 10) were conducted to locate the sources of these differences. The tests for seniority showed that doctors with less than 5 years’ experience had significantly lower knowledge indices than all the other groups, but that there were no significant differences between the other groups.

**Table 9:** Mann-Whitney tests of differences in doctors’ knowledge indices across seniority groups.

SENIORITY	Mean rank	Mann-Whitney U
Less than 5 years	65.3	U = 2085.5, p < .05
From 6 to 15 years	81.3	
		
Less than 5 years	68.8	U = 2288.5, p < .05
From 16 to 25 years	85.9	
		
Less than 5 years	78.0	U = 2836.0, p < .01
Over 26 years of seniority	100.0	
		
From 6 to 15 years	95.2	U = 4439, p = .96
From 16 to 25 years	94.8	
		
From 6 to 15 years	107.9	U = 5614.5, p = .9
Over 26 years of seniority	108.9	
		
From 16 to 25 years	112.0	U = 6140.5, p = .84
Over 26 years of seniority	113.8	

**Table 10:** Mann-Whitney tests of differences in doctors’ knowledge indices across professional status groups.

PROFESIONAL STATUS	Mean rank	Mann-Whitney U
Intern and Resident	39.7	U = 737.5, p = .16
Non-specialist doctor and specialist doctor under the training	47.0	
		
Intern and Resident	127.4	U = 4850.0, p < .01
Specialist doctor	170.2	
		
Intern and Resident	30.5	U = 304.5, p = .67
Other	32.8	
		
Non-specialist doctor and specialist doctor under the training	144.9	U = 4766.5, p = .28
Specialist doctor	161.5	
		
Non-specialist doctor and specialist doctor under the training	27.0	U = 244.5, p = .65
Other	25.0	
		
Specialist doctor	148.6	U = 1638.5, p = .28
Other	124.5	

The tests for professional status showed only one significant difference, specialist doctors having better knowledge than interns and residents.

### The problem of transparency of physicians’ prescriptions

Polish law states that pharmacists may not provide information about prescriptions made by physicians [21]. Violation of this provision has serious consequences for a pharmacist. Data from pharmacies are collected by IMS Health, which only presents data in aggregate rankings, making it difficult to deduce which medication was prescribed by a particular doctor. Only a physician may inform a representative about the prescriptions they make, but they do not have to do so. Nevertheless, as many as 61.5% of the surveyed physicians thought that PSRs would know what type of medications they prescribed, although only 9.8% declared that they had informed representatives themselves in this regard. Most respondents (67.4%) thought that PSRs had illegal ways of obtaining information from pharmacists, and 19.2% believed that market research firms provided PSRs with such information.

### Gift-giving

**Fig 2:**
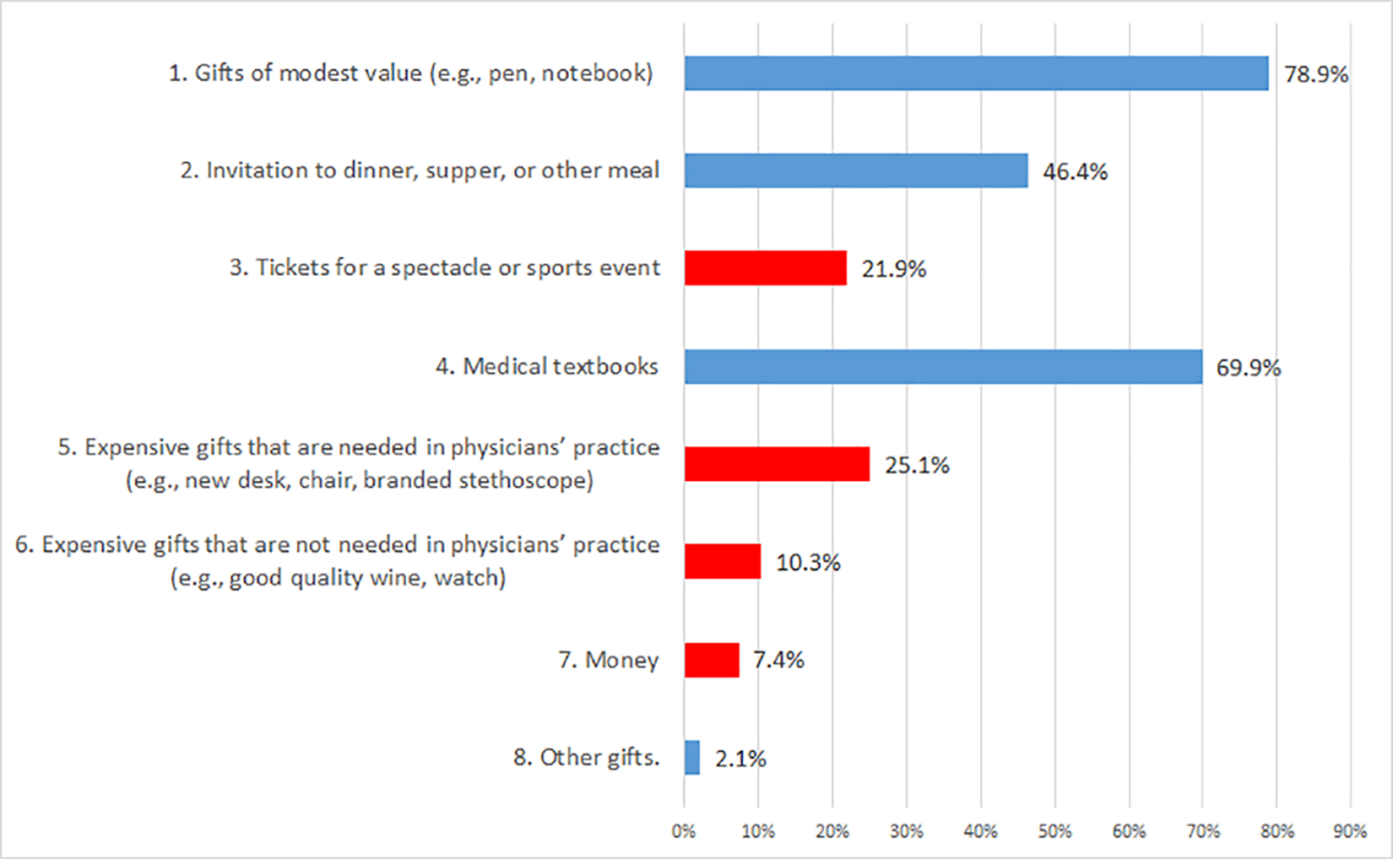
Gifts received by Polish physicians from pharmaceutical companies. Note. Percentages do not add up to 100% because respondents could select all applicable responses. Grey bars indicate gifts that are in accordance with Polish law, black bars indicate illegal gifts.

Doctors were asked about their knowledge of gifts received by other physicians from pharmaceutical companies. Most said that other doctors received small gifts (78.9%) and medical books (69.9%). Almost half of the respondents (46.4%) said that physicians accepted lunch invitations, dinners or other meals. All the aforementioned gifts are permissible according to Polish law. However, one quarter of respondents (25.1%) also indicated that doctors accepted expensive gifts related to their practice, and one in ten respondents (10.3%) admitted that doctors also received expensive gifts unrelated to their practice. Further, as many as 7.4% of respondents said that doctors also received money. Among other gifts mentioned by respondents were conference fees, spa vouchers, and brochures. One respondent also mentioned intimate contacts between representatives and doctors (see Fig 2 for a graphical representation of these data). More than one third of respondents (36.4%) indicated that they thought that physicians received at least one gift which is non-permissible under Polish law.

Finally, 12.1% of respondents agreed with a statement saying that their prescriptions were influenced by pharmaceutical companies’ gift-giving, and 28.7% agreed with a statement saying that their colleagues’ prescriptions were influenced by gifts from such companies. Thus, the doctors surveyed seemed to have perceived a greater impact of gifts on the decisions of other doctors than on their own decisions. Similar results were obtained in a 2008 study and this result is also consistent with much other research [11,12].

## Discussion

The present data demonstrate that the Polish physicians surveyed had extensive contacts with the pharmaceutical industry. The main research question posed in this article was: in what ways do Polish physicians cooperate with the pharmaceutical industry and what are the reasons for this? The majority of Polish physicians surveyed usually met with PSRs (85.0%). There were statistical associations showing that females did this more often than males, and physicians from smaller villages and cities did it more than those from larger cities. Another important way of cooperating was participation as an audience member in a conference, presentation, meeting, or educational training event organized by a pharmaceutical company. As many as 81.9% of physicians attended such educational meetings at least once during the 12 months preceding the survey. The majority of physicians (86.2%) received drug samples from PSRs. Physicians also received other gifts from the pharmaceutical industry. The most common gifts were small items such as pens, notebooks, and coffee. This is problematic since research shows that even small gifts can influence physicians’ prescription habits [13,14]. Because cooperation between Polish physicians and pharmaceutical companies is common and many gifts are received (even when they are not seen as gifts: drug samples, conferences fees, etc.) it is likely that Polish physicians’ prescribing habits are influenced by the notion of reciprocity. Further research is needed to test whether this is the case because if doctors do not prescribe drugs independently there may be conflicts of interests whereby the interests of patients are opposed to those of pharmaceutical companies and physicians.

For the most part, physicians did not acknowledge the above influences on their own behavior. Only 12.1% of physicians agreed with the sentence ‘Gifts from pharmaceutical companies influence my prescribing habits’ but more (28.7%) agreed with the sentence 'Gifts from pharmaceutical companies influence physicians’ prescribing habits’. So, as with their colleagues abroad [11, 12], they were more likely to perceive the pharmaceutical industry as influencing others than themselves.

The most commonly cited reasons for meeting with PSRs were: understanding of the difficulty of a PSR’s job (51.2%), the conviction that PSR’s are a valuable source of knowledge (49.9%), and the possibility of receiving funding for conference training (29.6%). Such answers evoke concerns about the independence of Polish physicians’ knowledge. Almost half of respondents admitted that pharmaceutical companies produced one of the three main sources of information about the drugs they used. We can therefore conclude that much of the knowledge of a large number of doctors is under the control of the pharmaceutical industry. Research has shown that information provided to physicians by the industry is often biased [7,9]. This is a threat to public health since many doctors may have incorrect knowledge about the medicines they prescribe to their patients owing to their use of information provided by pharmaceutical marketers. Because they are unaware of the strong effects of reciprocity (even the smallest gifts, including samples, may influence their prescriptions) they can constitute an easy target for PSRs, this being likely to sometimes result in their patients failing to obtain the best drugs for their ailments. This would compromise both the interests of the patient and the state (e.g., in the case of reimbursed drugs).

Furthermore, the surveyed doctors’ knowledge of the legal restrictions on physician–pharmaceutical industry cooperation was poor. An unexpected result of the study was that as much as 99% of physicians did not know how many samples of one product they were allowed to receive during one year.

One of the most interesting results was a significant difference in knowledge between respondents of differing seniority, as those working less than five years had little correct knowledge about the legal aspects of cooperation with the pharmaceutical industry and knowledge rose as seniority increased. This suggests that physicians learned about these contacts at work, during practice, and not at medical school. This implies that changes are necessary in the Polish medical school curriculum.

Another interesting result was the one whereby, despite a legal ban, 35% of doctors said they usually met with representatives in their offices during working hours, and another 31.8% declared that, although they only met with PSRs during breaks, they still met them in their offices. This shows that Polish laws aimed at limiting physicians’ meetings with PSRs during working hours, and reducing patient waiting times because of such appointments, are not functioning properly, and overall the present results show that Polish doctors require training about the current legal restrictions on their cooperation with the pharmaceutical industry.

The present research did not answer all questions arising from cooperation between physicians and the pharmaceutical industry. Future research should investigate the extent to which prescriptions written by Polish physicians are dependent upon their contacts with the pharmaceutical industry. Among other things, such research might make doctors change their attitudes toward pharmaceutical marketing and be more open to education in this area: the study showed that most of them (87.9%) were convinced that they were resistant to industry influences. There is also a need for in-depth qualitative studies, since the present questionnaire approach provided only superficial information. Such studies could be conducted using focus group interviews so that doctors’ own attitudes (as opposed to those preconceived by researchers) are allowed to emerge during discussions.

The greatest weakness of this research was that the sample obtained was non-representative resulting in the possibility that the results may not be generalizable to the population of Polish physicians. A larger scale study would rectify this. Nevertheless, the main strength of study is that it has provided some initial information on an important issue which has previously been neglected in Poland. Also, the questionnaire approach permitted the assessment of a wide range of aspects of the physician–pharmaceutical industry relationship.

## Conclusions

Cooperation between doctors and the pharmaceutical industry has interested researchers, journalists and patients for many years. The conflicts of interest that can arise through such cooperation lead to a number of risks to public health, and authorities responsible for developing public health policy should be interested in creating better transparency of this relationship.

The present study has shown that the pharmaceutical industry influences Polish physicians in various ways: organizing their meetings with PSRs, organizing conferences, giving them the possibility of earning money (e.g., through phase IV research), gift-giving, etc. Also, the present findings give a particular cause for concern in that Polish physicians often seem to rely on information from the pharmaceutical industry.

Various solutions to the problems presently identified might be suggested. First, doctors should be educated in the many different aspects of cooperating with the pharmaceutical industry from the beginning of their studies. Second, physicians should have cyclical training about legal and ethical aspects of cooperation with the pharmaceutical industry. Third, doctors need to be made to disclose any possible conflicts of interest to create full transparency. Fourth, physicians should be discouraged from participating in educational meetings organized by the industry and discouraged from accepting gifts of any nature, even drug samples. The results of the current study indicate that Polish medical society needs a greater awareness of the necessity for these changes.

## Supporting information

S1 File Questionnaire form. (DOC)
