# Supplementary material for: Polish physicians’ cooperation with the pharmaceutical industry and its potential impact on public health
Source: PLoS One. 2017 Sep 19;12(9):e0184862. doi: 10.1371/journal.pone.0184862 (PMC5604986; doi:10.1371/journal.pone.0184862)
Supplement: S1 File — (DOC) [file pone.0184862.s001.doc]

Dear Sir or Madam,

My name is Dr Marta Makowska. I am an Assistant Professor in the Sociology Department at Warsaw University of Life Sciences. I have conducted research into different aspects of pharmaceutical marketing to physicians for many years. Neither myself nor my research are associated with pharmaceutical companies in any way.

The questionnaire that you have in front of you is almost identical to one that I used to conduct research five years ago with the help of Medycyna Praktyczna and it aims to examine Polish physicians’ attitudes toward pharmaceutical marketing.

The questionnaire is anonymous: data collected using the Internet portal are adjusted to remove any information that could be used to identify respondents, including their computer’s IP address. All replies will be analyzed and presented only in the form of collective statistical data sets at scientific conferences and in articles. Your frank replies are very important for my research because they will enable accurate evaluation of physicians’ approaches to medical sales representatives and their activities.

The survey should take you no more than 10 to15 minutes. Please complete the questionnaire as a whole, do not skip questions; this will be very important during the analytic phase of the study. To go to the next page, click "Next Page".

If you prefer to fill in the questionnaire in a different format, for example, to receive it directly via email, traditional post or any other way, please contact me at: marta_makowska@sggw.pl. I will try to answer as soon as possible.

To complete the questionnaire please go to: (the link was here).

Thank you very much for help!

Dr. Marta Makowska

(Q1) During your medical practice have you ever met with a medical sales representative from a pharmaceutical company?

1. Yes
2. No (go to Q3)

(Q2) Do you usually meet with medical sales representatives?

1. Yes (go to Q4)
2. No

(Q3) Why do you not meet with pharmaceutical sales representatives? Please select up to 3 main reasons. (go to D1)

1. I do not have time for them
2. I do not want to be accused of corruption
3. It is not in patients’ interests
4. It is prohibited at the institution where I work
5. I do not trust what they say
6. I trust drugs that I have used for a long time
7. I have confidence in my own discernment with respect to drugs
8. I believe that medical sales representatives are rude
9. Medical representatives do not visit me
10. Other reasons - please say what they are ...

(Q4) Where do you meet with pharmaceutical sales representatives?

1. In my office/workplace during work time
2. In my office/workplace after work
3. In my office/workplace during a break at work
4. Outside my office/workplace during work time
5. Outside my office/workplace after work
6. Outside my office/workplace during a break at work

(Q5) How many minutes did the last visit of a pharmaceutical sales representative take?

_______ mins.

(Q6) How many pharmaceutical sales representatives visit you per week on average?

_____________

(Q7) What are the reasons that you meet with medical sales representatives? Please select up to 3 main reasons.

1. I think that the information they provide about drugs is valuable
   2. Thanks to them, I have the opportunity of earning extra money (e.g., White Saturdays, completing questionnaires)
   3. Thanks to them, I have the possibility of receiving funding for conferences, training, etc.
   4. If I do not let them in they will stand at the office door
   5. I am unable to refuse them
   6. I accept them because I understand their job
   7. Thanks to them, I have a break between patients’ visits
   8. I like the gifts they offer
   9. They provide me free samples of drugs
   10. Meeting with medical sales representatives is an obligation, a part of working in the medical profession
   11. I have no time for the self-study of information about medicines
   12. Other reasons - please say what these are ....

(Q8) Do you see a difference between medical sales representatives who promote generic drugs and medical sales representatives who promote innovative drugs?

1. Yes
2. No (go to Q11)

(Q8_1) What is the difference?

………………………………….

(Q9) In your opinion, what is the most important function of pharmaceutical sales representatives from a generic company? Please select only one answer.

1. Promoting and advertising drugs
   2. Selling drugs
   3. Cooperation with a doctor, helping to choose the most appropriate treatment for patients
   4. Educating, providing information about medicines and their use
   5. Other functions - please say what they are ....

(Q10) In your opinion, what is the most important function of pharmaceutical sales representatives from an innovative company? Please select only one answer

(go to Q12).

1. Promoting and advertising drugs
2. Selling drugs
3. Cooperation with a doctor, helping to choose the most appropriate treatment for patients
4. Educating, providing information about medicines and their use
5. Other functions - please say what they are ....

(Q11) In your opinion, what is the most important function of pharmaceutical sales representatives? Please select only one answer.

1. Promoting and advertising drugs
2. Selling drugs
3. Cooperation with a doctor, helping to choose the most appropriate treatment for patients
4. Educating, providing information about medicines and their use
5. Other functions - please say what they are ....

(Q12) What sources of information about drugs do you most frequently use? Please choose up to 3 sources.

1. Medical books
2. Medical journals
3. Drug leaflets
4. Leaflets and other materials from pharmaceutical companies
5. The Internet
6. Meetings and training courses organized by pharmaceutical companies
7. Scientific conferences
8. Medical sales representatives
9. Other sources - please say what they are ....

(Q13) Do you trust the reliability of the information provided by medical sales representatives?

1. Definitely yes *(go to Q15)*
2. Mostly yes *(go to Q15)*
3. Mostly no
4. Definitely no

(Q14) Why do you not trust the information given by medical representatives? Please select one main reason.

1. They often do not have medical or pharmaceutical training
2. A representative has previously given me incorrect information
3. They are salespeople and their main objective is to sell the product
4. Pharmaceutical companies manipulate information about their medicines
5. A different reason - please say what this is ....

(Q15) Do you receive samples of drugs from medical sales representatives?

1. Yes

2. No (go to Q17)

(Q16). What do you usually do with drug samples received from medical sales representatives?

1. I give them patients to start treatment
2. I give them to my poorest patients who cannot afford drugs
3. I distribute them among medical staff
4. I use them for myself, my family and friends
5. I do not do anything with them, they lie in the office
6. I use them differently - please say how ....

(Q17) How many boxes of free samples of the same drug do you think a representative should offer a medical doctor in a year?

_______

(Q18) Do you think that medical sales representatives know what medications you prescribed to patients?

1. Yes, they know
2. No, they do not know *(go to Q20)*

(Q19) How do medical sales representatives know what drugs you prescribe? Please indicate the main source.

1. I tell them
2. They have ways of getting information from pharmacists in pharmacies
3. Representatives interview patients at the office
4. Market research firms provide them with such information
5. Other sources - please say which ....

(Q20) How do you rate the ethics of medical sales representatives?

1. Definitely high
2. Rather high
3. Rather low
4. Definitely low

(Q21) Please indicate the methods through which you have contact with pharmaceutical sales representatives.

1. Talk with medical sales representatives by phone
2. Talk with representatives by internet with video (for example, Skype)
3. Talk with representatives by internet without video (for example, Chat)
4. SMS from medical sales representatives
5. Mail from medical sales representatives

(Q22) Which form of contact with sales representatives is best for you?

1. Face-to-face talk with medical sales representatives
2. Talk with medical sales representatives by phone
3. Talk with representatives by internet with video (for example, Skype)
4. Talk with representatives by internet without video (for example, Chat)
5. SMS from medical sales representatives
6. Mail from medical sales representatives
7. Other form, please say which …

(Q23) As far as you know, what gifts do physicians receive from pharmaceutical sales representatives? Choose as many answers as necessary.

1. Gifts of modest value (e.g., pen, notebook, flowers, coffee, tea, chocolates).
2. Invitations to dinner, supper, or other meals.
3. Tickets for sports events or other spectacles.
4. Medical textbooks.
5. Expensive gifts that are needed for practice (e.g., new desk, chair, brand electronic stethoscope).
6. Expensive gifts that are not needed for practice (e.g., good quality wine, watch).
7. Money.
8. Other, please say what ...

(Q24) During last 12 months did you:

|  | **Yes** | **No** |
| --- | --- | --- |
| 1. Participate as a speaker in a lecture, seminar, presentation or other educational meeting organized by a pharmaceutical company. | 🞐 | 🞐 |
| 2. Participate as a listener in a lecture, seminar, presentation or other educational meeting organized by a pharmaceutical company. | 🞐 | 🞐 |
| 3. Participate in the post-marketing research of a drug organized by a pharmaceutical company (Phase IV trials). | 🞐 | 🞐 |
| 4. Participate in research organized by a pharmaceutical company into the assessment of pharmaceutical sales representatives’ work. | 🞐 | 🞐 |
| 5. Investigate patients while hired by a pharmaceutical company (e.g., White Saturday, White Sunday). | 🞐 | 🞐 |
| 6. Write or authorize any articles about an active substance or drug at the request of a pharmaceutical company. | 🞐 | 🞐 |

(Q25) Please say how much you agree or disagree with each of the following statements:

|  | | **1**  **Strongly agree** | | **2**  **Somewhat agree** | | **3**  **Somewhat disagree** | | **4**  **Strongly disagree** |
| --- | --- | --- | --- | --- | --- | --- | --- | --- |
| 1. Gifts to physicians from pharmaceutical companies are also beneficial to patients. | | 🞐 | | 🞐 | | 🞐 | | 🞐 |
| 2. Gifts from pharmaceutical companies influence my prescribing habits. | | 🞐 | | 🞐 | | 🞐 | | 🞐 |
| 3. Physicians should have the right to take gifts of modest value from pharmaceutical companies which are needed in their practice (e.g., pen, notebook, flowers, coffee, tea, chocolate). | | 🞐 | | 🞐 | | 🞐 | | 🞐 |
| 4. Physicians should have the right to take expensive gifts from pharmaceutical companies if they are needed in their practice (e.g., a new desk, chair, branded stethoscope). | | 🞐 | | 🞐 | | 🞐 | | 🞐 |
| 5. Physicians should have the right to take gifts from pharmaceutical companies in any form in which companies are likely to give them (e.g., good quality wine, watch). | | 🞐 | | 🞐 | | 🞐 | | 🞐 |
| 6. Sometimes during visits a representative does not even mention a word about the drugs they are promoting. | | 🞐 | | 🞐 | | 🞐 | | 🞐 |
| 7. Physicians should have the right to perform paid work for pharmaceutical companies (e.g., completing questionnaires or examining patients). | | 🞐 | | 🞐 | | 🞐 | | 🞐 |
| 8. Drug samples are also gifts from pharmaceutical companies. | | 🞐 | | 🞐 | | 🞐 | | 🞐 |
| 9. Physicians should have the right to obtain support from pharmaceutical companies to attend conferences, training courses, etc. | | 🞐 | | 🞐 | | 🞐 | | 🞐 |
| 10. Gifts from pharmaceutical companies are rewards for prescribing their medicines. | | 🞐 | | 🞐 | | 🞐 | | 🞐 |
| 11. Gifts from pharmaceutical companies influence physicians’ prescribing habits. | | 🞐 | | 🞐 | | 🞐 | | 🞐 |
| 12. My relationships with certain medical representatives are friendly. | 🞐 | | 🞐 | | 🞐 | | 🞐 | |
| 13. I only accept sales representatives from chosen companies. | 🞐 | | 🞐 | | 🞐 | | 🞐 | |

**DEMOGRAPHICS:**

(D1) Please indicate your gender.

1. Female
2. Male

(D2). Please indicate your year of birth.

________

(D3) Please indicate the year you started work as a physician.

________

(D4) Please indicate your occupational status.

1. Intern
2. Resident
3. Non-specialist doctor
4. Specialist doctor under training
5. Specialist
6. Other, please say what...............

(D5) Where do you currently work? Please indicate all places.

1. Public hospital
2. Non-public hospital
3. Public clinic
4. Non-public clinic
5. Office, clinic or hospital without a contract with the National Health Fund
6. Emergency
   7. Other, please say what………

(D6) Have you ever work as a pharmaceutical sales representative?

1. Yes
2. No

(M7) Please indicate the size of the place you currently live.

1. A village
2. A city of up to 20 thousand residents
3. A city with between 21 thousand and 200 thousand residents
4. A city with between 201 thousand and 500 thousand residents
5. A city with over 500 thousand residents

**Thank you for completing the questionnaire!**

If you have any additional remarks about the questionnaire or you want share your opinion about pharmaceutical marketing, please do so in the space below.
